# Survival rates of cervical cancer patients in Sarawak: a single-centre referral study

**DOI:** 10.1186/s12885-025-14678-9

**Published:** 2025-08-27

**Authors:** Melissa Siaw Han Lim, Shirley Siang Ning Tan, Izzati Binti Wan Maharuddin, Pei Jye Voon, Nur Khairiyah Binti Abdul Rahim, Xun Ting Tiong, Cassandra Sheau Mei Chee, Adam Malik Ismail, Yolanda Augustin

**Affiliations:** 1 Faculty of Medicine and Health Sciences, UNIMAS, Kota Samarahan, Malaysia; 2 Clinical Research Centre, Institute for Clinical Research, Sarawak General Hospital, National Institutes of Health, Ministry of Health, Kuching, Malaysia; 3 Department of Pharmacy, Sarawak General Hospital, Ministry of Health, Kuching, Malaysia; 4 Department of Radiotherapy, Oncology and Palliative Care, Sarawak General Hospital, Ministry of Health, Kuching, Malaysia; 5 Department of Gynaecology, Sarawak General Hospital, Ministry of Health, Kuching, Malaysia; 6 Department of Pathology, Sarawak General Hospital, Ministry of Health, Kuching, Malaysia; 7 School of Health and Medical Sciences, Institute of Infection and Immunity, City St George’s University of London, London, UK; 8 UM Affordable Diagnostics and Therapeutics, Department of Pharmacology, Faculty of Medicine, Universiti Malaya, Kuala Lumpur, 50603 Malaysia

**Keywords:** Cervical cancer, Sarawak, Epidemiology, Survival analysis

## Abstract

**Background:**

Cervical cancer represents a significant health challenge in Malaysia, especially in the state of Sarawak which records some of the highest incidence rates across the country. This study evaluates the survival rates of cervical cancer patients in Sarawak, focusing on demographic characteristics, disease stage and survival outcomes to inform healthcare strategies.

**Methods:**

A retrospective case notes review of disease stage, patterns of care and survival outcomes of patients diagnosed with cervical cancer at Sarawak General Hospital between January 2018 to December 2022 was conducted. Kaplan-Meier survival analysis and Cox Regression analysis were performed to assess survival outcomes and factors influencing survival.

**Results:**

A total of 555 patients were included in this review. The majority of patients were diagnosed between the ages of 40 and 59, with a mean age of 53 years. Ibans comprised the largest subgroup by ethnicity. Only 11.2% of patients were diagnosed with Stage I disease. The majority of patients were diagnosed at advanced stages III and IV. The overall 5-year survival rate was 59.4%. Factors significantly affecting survival included FIGO cancer stage and ethnicity.

**Conclusions:**

Diagnosis at advanced stages of disease lead to poorer clinical outcomes in cervical cancer patients in Sarawak. This study highlights the critical need for enhanced screening and early diagnosis to improve survival rates amongst cervical cancer patients Sarawak. Efforts should focus on improving cervical health literacy, expanding access to healthcare services and improving the uptake of HPV vaccination and cervical screening particularly in rural communities.

## Introduction

Cervical cancer is a major cause of morbidity and mortality amongst women, with 600,000 cases and 341,000 deaths recorded globally in 2020 [1, 2]. Low-and-Middle-Income-Countries (LMICs) are disproportionately affected with 80% of cases and 90% of cervical cancer-related deaths occurring in low resource settings.

In Malaysia, cervical cancer was the third most common cancer among Malaysian women in 2020 and a leading cause of mortality among Malaysian women aged 15 to 44 [3]. Malaysian women between the ages of 30–69 years old died from cancer at a rate of 73.3 per 100,000 population, similar to ischemic heart disease at 75.8 per 100,000 population [4]. Around 64% of all cancer cases presented late at Stages III and IV [5]. In 2018, 1,682 women were diagnosed with cervical cancer, and 944 died from the disease [6]. The state of Sarawak recorded amongst the highest incidence of cervical cancer in Malaysia with an incidence of 12.1 per 100,000 women [7, 8].

Sarawak is the largest state in Malaysia with a population of around 2.9 million, the majority of whom still live in rural and semi-rural settings [4]. Patients often have to travel many hours and cover hundreds of kilometres to reach district general hospitals or the main cancer unit at Sarawak General Hospital in the state capital of Kuching. These communities are at risk of delayed diagnosis due to limited access to screening and reduced cervical health literacy and are often diagnosed with advanced disease as a consequence [8].

Despite the proven effectiveness of cervical cancer screening and HPV vaccination, worldwide coverage of these preventive measures remain poor, especially in LMICs [9, 10]. In May 2018, the World Health Organisation (WHO) Director-General announced a global call to action to eliminate cervical cancer with three key pillars: a 90% HPV vaccination rate, 70% screening, and 90% treatment [11]. Malaysia began its national HPV vaccination programme in 2010, offering free HPV vaccines to 13-year old girls through a school-based immunization initiative. Pre-COVID, the HPV vaccination rate among females ages 13 was 84.5% (completed 2 doses), but there were no published data on vaccination uptake according to individual states [12]. However this programme was disrupted during the COVID-19 pandemic due to pandemic lockdowns and a shortage of HPV vaccination supply, which the Ministry of Health is now addressing through catch up vaccination. In Sarawak, children from some ethnic communities may leave school early due to socio-economic factors, which may result in them missing out on the national HPV vaccination programme. Cervical screening uptake in Malaysia remains low, with less than 30% of the eligible population participating in screening nationally [9]. At present there is no published data on vaccination or screening uptake by state. A number of studies have shown that barriers to cervical screening include lack of awareness and knowledge of screening as well as embarrassment [13–17]. Although Sarawak has amongst the highest incidence of cervical cancer nationwide, there is a lack of local data on the biological and socio-economic determinants of cervical cancer risk in the local population. There is a need for robust data on cervical cancer in Sarawak to inform national Clinical Practice Guidelines (CGP) as data from Sarawak cervical cancer cohorts remains lacking [7].

In this study, we aim to report survival rates of cervical cancer patients in Sarawak over a 5 year period between 2018 and 2022. We also aim to identify the specific factors associated with survival in a multi-ethnic population equipped with only one referral centre in Sarawak, Borneo. Findings from this real world study of cancer staging, patterns of care and survival outcomes of patients in Sarawak will inform gaps and challenges that need to be addressed to increase early detection and improve patient survival.

## Materials and methods

### Data design, setting and management

We conducted a retrospective case notes review of cervical cancer patients diagnosed at Sarawak General Hospital between January 2018 to December 2022. Medical records were obtained from the Department of Radiotherapy, Oncology and Palliative Care, Sarawak General Hospital (RTU) which is the only public tertiary hospital equipped with surgery, radiotherapy, chemotherapy and palliative care facilities in Sarawak. Patients with missing information such as date of diagnosis, those diagnosed before 2018 and duplicate profiles were excluded. The survival status of the patients (alive or deceased) at the end of the study period and date of death were obtained by examining the mortality data from the National Registration Department (JPN).

### Patient characteristics

Factors considered in the analysis of this study were age and stage at diagnosis, ethnicity, histology and primary oncological treatment. Cancer staging followed the 2018 International Federation of Gynaecology and Obstetrics (FIGO) system [18]. All patients were diagnosed based on a confirmed cervical biopsy report. Pre-treatment staging computed tomography (CT) scans were performed for all patients, and treatment decisions were guided by the FIGO stage.

For patients diagnosed with Stage I disease, the primary treatment was surgery, followed by adjuvant radiation therapy or chemoradiation depending on the presence of pathological risk factors. For patients with Stage II to Stage IVA disease, the standard treatment was concurrent chemoradiation. This included external beam radiation therapy (EBRT) to the pelvis (+/- para-aortic nodes), administered at a dose of 45 to 50.4 Gy in 25 to 28 fractions. Concurrent chemotherapy consisted of weekly cisplatin (40 mg/m²) or carboplatin (AUC 1.5 to 2) for five cycles.

EBRT was delivered using three-dimensional conformal radiotherapy (3D-CRT), with a sequential boost of 8 to 16 Gy to involved pathological lymph nodes. Following EBRT, patients underwent intracavitary brachytherapy without the use of interstitial needles. Brachytherapy was delivered in three fractions using a conventional 2D technique, with each fraction delivering 7 Gy, using high-dose-rate Iridium-192.

For patients with Stage IVB disease, treatment decisions were individualized based on baseline performance status and comorbidities. Options included multimodal approaches with systemic chemotherapy as the first-line treatment, and palliative care or no active treatment depending on clinical considerations.

### Statistical analysis

Statistical analyses were performed using SPSS v17.0 and also R program. Continuous variables were presented in mean ± standard deviation or median (IQR) as appropriate, and categorical variables as percentages. The duration of events were expressed in months. Kaplan Meir survival analyses were conducted to determine the overall survival and median survival between different cancer stage. COX Regression analyses were performed to determine factors associated with survival. A p value less than 0.05 demonstrated statistical significance (two tailed).

## Results

### Demographic data

A total of 590 patients were initially identified. Of these, 555 patients were included in a single cervical cancer database, with 35 excluded based on pre-specified exclusion criteria. The mean age was 53.29 ± 12.4 years old. Approximately one third of the patient population was made up of patients aged 60 years and above (*n* = 180;32.4%), followed by age group 50–59 (*n* = 159; 28.6%) and age group 40–49 (*n* = 136; 24.5%). Only 14.4% of patients were under 40 years of age. The ethnic composition was predominantly indigenous Iban (*n* = 201; 36.1%), followed by Chinese (*n* = 123; 22.2%), Malay (*n* = 103 patients; 18.6%), Bidayuh (*n* = 39; 7%), Melanau (*n* = 23; 4.1%) and other ethnic groups (*n* = 68; 12.2%).

Only 43 patients (7.8%) reported a history of smoking. A total of 332 patients (59.9%) were classified as B40 (bottom 40% of households in Malaysia, classified by household income level) (results not shown).

A total of 177 subjects had duration of symptoms clearly recorded, with the median duration from onset of symptoms to initial diagnosis reported as 8 months. The majority of the patients (*n* = 423;74.7%) reported intermenstrual/postmenopausal/abnormal discharge as a presenting symptom. An abnormal pap-smear was the reason for the initial referral in only 12(2.2%) of cases. The majority of patients (*n* = 549; 98.9%) did not have record of HPV vaccination status, with the majority not having been eligible for the national screening vaccination programme based on their age. Most women (*n* = 531; 95.7%) were married or had previously been married.

At the time of diagnosis, only 63 patients (11.4%) were diagnosed with Stage I cervical cancer according to the FIGO cervical cancer classification [18]. The majority of patients were diagnosed with stage II (*n* = 185; 33.5%) and stage III (*n* = 179; 32.4%) disease. A total of 126 patients (22.8%) presented with stage IV disease at diagnosis.

The median duration from initial diagnosis to starting first treatment was 1.7 months. The median duration from referral to first appointment at the Radiotherapy Unit (RTU) was 0.3 months, whereas duration from first appointment at RTU to starting first treatment was 0.5 months.

The majority of patients were treated with both chemotherapy and radiation (*n* = 316; 57.0%). Around a fifth of patients were treated with radiation alone (*n* = 109; 19.7%). A total of 53 patients (9.4%) were treated with surgery ± chemotherapy ± radiation. A total of 48 patients (8.6%) had chemotherapy only while 28 patients (5.0%) did not have any treatment recorded.

From the record of 133 patients, the median follow-up duration time calculated from diagnosis to the last recorded visit date at RTU, was 9.3 months (range: 0–62.0 months).

### Histology subtypes

The majority of the patients were diagnosed with squamous cell carcinomas (*n* = 409; 73.7%), followed by adenocarcinomas (*n* = 123; 22.2%). A small proportion were diagnosed with adenosquamous carcinomas (*n* = 5; 0.9%) and other histological classifications which include neuroendocrine tumors, melanoma, and germ cell tumor (*n* = 18; 3.2%).

### Referring institutions

Most patients were referred from government or public health hospitals and clinics across Sarawak. A small proportion (*n* = 69; 12.5%) were referred from private hospitals.

### Survival analysis

Patient survival data was obtained from the National Registration Department (JPN) database to check for cause-of-death and date-of-death up to 30th June 2024. The median survival follow-up time– calculated from diagnosis to the date of death or censoring, was 27.1 months (range: 0.2–77.7 months). A total of 186 deaths were recorded (33.5%). Patients who were not recorded as deceased as of 30th June 2024 (*n* = 369) according to JPN record were censored. A survival curve was plotted on all-cause mortality vs. duration from initial diagnosis to death. As the specific cause of death was often documented as “unknown” it was not possible to report cancer specific mortality accurately. In a univariate COX regression analysis, both FIGO cancer staging and ethnicity were found to significantly affect cervical cancer survival. Both variables, in addition of age category, WHO histology, and treatment modality were inputted into a multivariate COX regression model. The observed overall survival rates at 1, 2, 3 and 5 years were 84.6%, 72.0, 66.1% and 59.5%, respectively. Overall, median survival was not achieved during the duration of follow up. However, when divided into stages, patients with stage IV disease, had a median survival time of 23.6 months (confidence interval ranging from 11.1 months to 36.1 months) and a 5-year survival rate of 38.0% (Stage IVA: 41.3%; Stage IVB: 32.7%). Patients initially presenting with Stage IV were 4.4 times more likely to die compared to subjects who were presented at Stage 1 (adjHR:4.4, *p* < 0.001). Chinese women had the highest overall survival rate at 67.0%, followed by Iban women with an overall survival rate of 58.5%. In contrast, Malay women had the lowest survival rate at 45.7%, with an adjHR of 2.0, *p* < 0.002. There was no significant difference in the proportion of cervical cancer stage at diagnosis between the different ethnic groups.

## Discussions

**Fig. 1:**
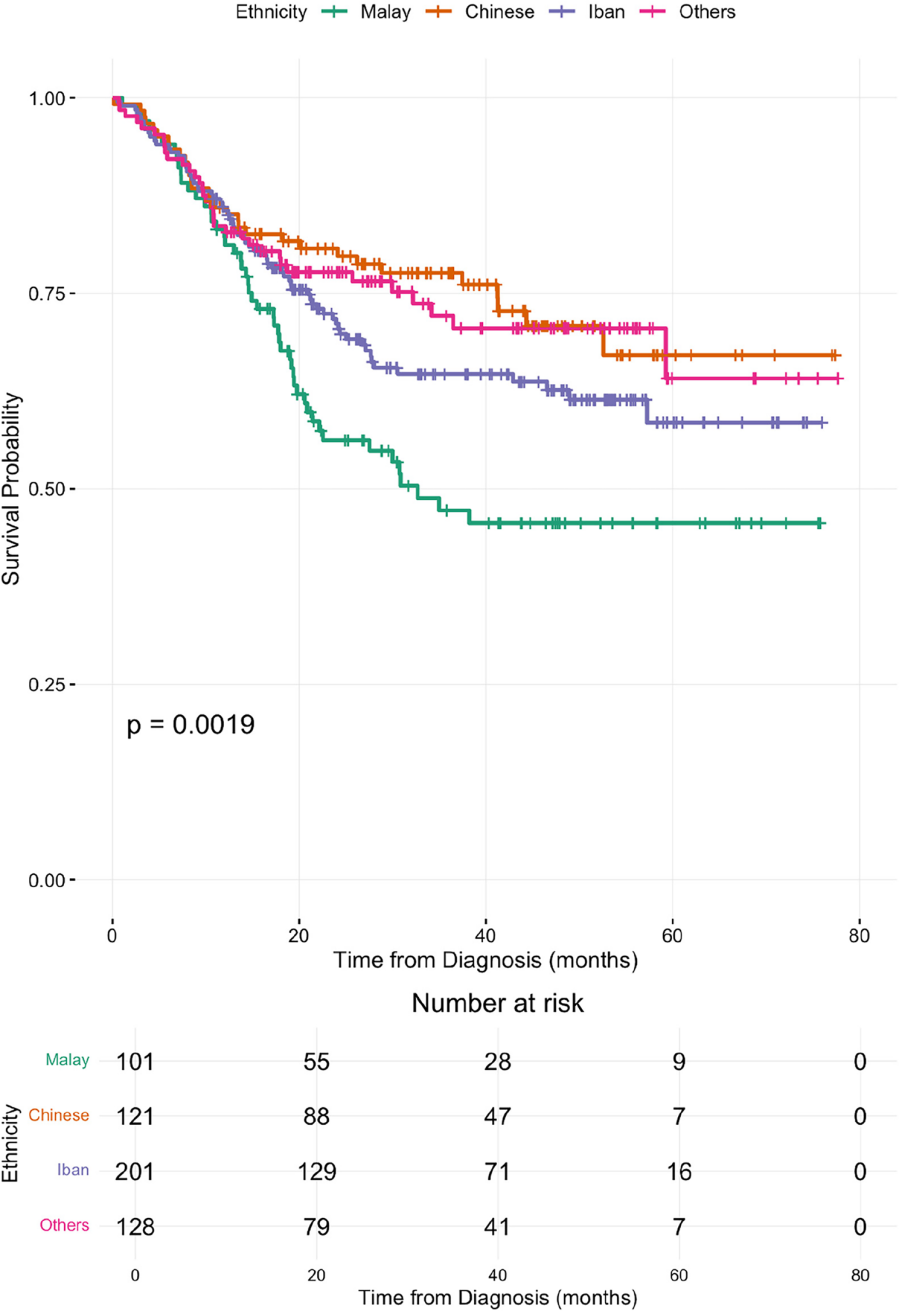
Ethnic groups and survival rate

This is the first study to publish patient demographics, diagnostic stage, patterns of care and survival outcomes in patients diagnosed with cervical cancer in Sarawak. Similar to a previous national study, the majority of cervical cancer patients were diagnosed between the age of 40 to 59, with a mean age of 53 years old [19]. Our patient cohort comprised 36.2% indigenous Iban, 22.2% Chinese and 18.6% Malay, a demographic unique to Sarawak, as the Iban are the largest indigenous ethnic group. A high proportion of Ibans live in rural settings, where travel distance and costs can be barriers to accessing cervical screening and healthcare services. In spite of this, Iban women recorded a relatively good overall survival rate of 58.5%. Chinese women had the highest overall survival rate at 67.0%. In contrast, as shown in Fig. 1, Malay women had the lowest survival rate at 45.7%, which is consistent with findings from a previous study [19]. There was no significant difference in the proportion of cervical cancer stage at diagnosis between the different ethnic groups. The reason for the difference in survival outcomes remains unclear but similar observations were highlighted by Muhamad et al., who discussed the peculiar health-seeking behaviour of Malay women. Many Malay women prefer traditional treatments over modern medical treatments [20]. They are also notorious at seeking treatment at a later stage of cancer and often presents with large tumours [14, 21]. However, this needs to be investigated further as no definitive conclusions can be drawn as cause of death and cancer specific survival outcomes were not available in many cases from the death registry and hence are not reported here. A prospective cancer registry that collects data on treatment completion rates, best response to treatment, cancer specific survival, multi-morbidity and cause of death is needed to gain a clearer picture of why overall survival outcomes appear to be worse amongst Malay women.

**Fig. 2:**
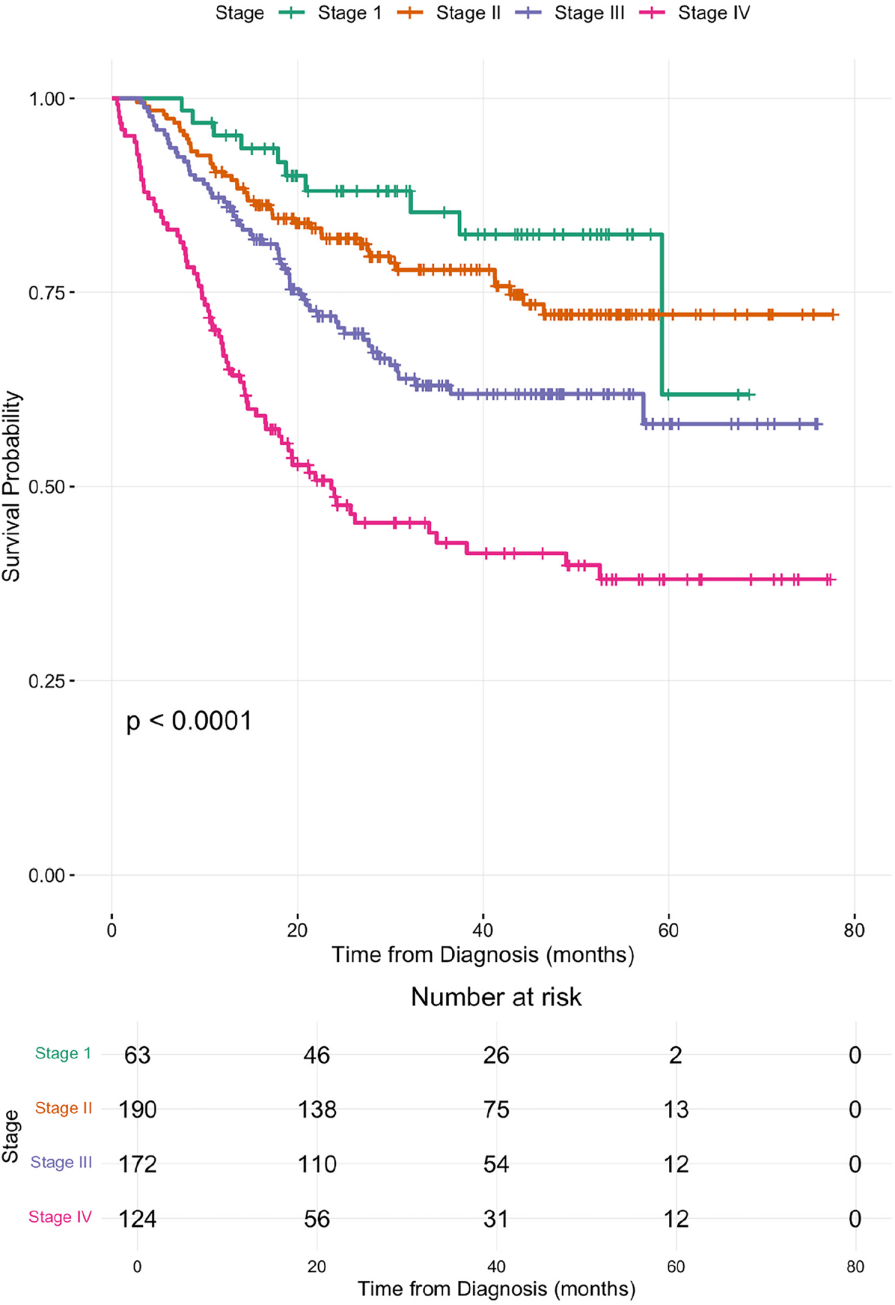
FIGO staging and survival rate

In our study, more than 50% of patients were diagnosed at late stages III and IV. Patients diagnosed at Stage IV had the lowest 5-year survival rate of 38.0%, compared to Stage III (58.1%), Stage II (72.2%) and Stage I (61.8%) (Fig. 2). Cervical cancer stages were determined by the International Federation of Gynaecology and Obstetrics (FIGO) staging system which categorised the cancer into stages according to tumour size [18]. FIGO staging has been the standard method for assessing survival rates in cervical cancer, where larger tumour size is associated with poorer disease control and, consequently, lower survival rates. Our study showed that although Stage II had a higher 5-year survival rate compared to Stage I, the difference was not statistically significant, *p* = 0.254 (Table 1). On the other hand, the 5-year survival rate for Stage IV in our study was 38%, which appears relatively higher than other Asian countries − 21.1% in Korea [22] and 0% in Indonesia [23, 24]. Patients with Stage IVA and IVB also appeared to have relatively higher survival rate of 41.3% and 32.7%, respectively, compared to those reported in other countries [25, 26]. This could be explained by the fact that this was a retrospective observational study, 5-year follow up is still ongoing for some cohorts. It is also possible that death statistics may not be fully reported in rural communities, particularly as this study also spanned periods of lock down during the COVID-19 pandemic.

For the histopathology factor, our study reported that patients with squamous cell carcinoma had lower 5-year survival rates compared to patients with adenocarcinoma (59.8% vs 60.9%, respectively), which is inconsistent with previous studies [27, 28], including a local study by Razak et al., which found that patients with squamous cell carcinoma had better survival than those with adenocarcinoma [26]. However, studies conducted in Indonesia reported that patients with squamous cell carcinoma had worse prognosis compared to those with adenosquamous carcinoma and adenocarcinoma [24, 29]. Therefore, our findings are more similar to those reported in Indonesia compared to our western Malaysian counterpart. More studies are warranted to delineate the relationship between histopathology subtype and cervical cancer survival rates in these regions. 

**Table 1:** Multiple COX regression analysis of factors associated with survival rate (%) among cervical cancer patients in Sarawak

	1 year (%)	2 year (%)	3 year (%)	5 year (%)	HR (95%CI)	*P* value	adjHR (95%CI)	*P* value
**Age**						0.140		0.034
< 30	72.7	51.9	51.9	51.9	2.1 (0.8–5.3)	0.110	1.9 (0.8–4.9)	0.167
30–39	85.5	66.5	64.2	64.2	1.2 (0.8-2.0)	0.403	1.5 (0.9–2.5)	0.146
40–49	88.9	74.9	67.9	60.6	1.1 (0.7–1.6)	0.784	1.0 (0.7–1.6)	0.882
50–59	80.5	65.9	60.9	53.2	1.5 (1.0-2.2)	0.032	1.7 (1.2–2.5)	0.007
>/=60 years (ref)	85.3	78.4	70.9	63.4				
**Ethnicity**						0.003		0.006
Malay	82.2	56.5	47.3	45.7	1.9 (1.2-3.0)	0.003	2.0 (1.3–3.1)	0.002
Chinese	85.9	80.7	77.6	67.0	0.9 (0.6–1.5)	0.670	1.1 (0.7–1.7)	0.844
Iban	85.5	71.1	64.8	58.5	1.3 (0.8–1.9)	0.275	1.3 (0.9-2.0)	0.174
Others (ref)	83.6	77.7	72.0	64.0				
**FIGO Staging**						< 0.001		< 0.001
Stage I (ref)	95.2	88.1	82.5	61.8				
Stage II	90.5	82.0	77.9	72.2	1.5 (0.8-3.0)	0.254	1.5 (0.7-3.0)	0.258
Stage III	87.2	72.0	63.0	58.1	2.4 (1.2–4.7)	0.010	2.4 (1.2–4.7)	0.013
Stage IV	66.8	48.5	42.7	38.0	5.0 (2.6–9.6)	< 0.001	5.3 (2.7–10.3)	< 0.001
Stage IVA	67.2	52.7	44.8	41.3				
Stage IVB	65.5	43.5	39.0	32.7				
**WHO Histology**						0.712		0.728
Squamous cell carcinomas (ref)	82.8	71.9	66.7	59.8				
Adenocarcinomas	90.9	74.8	66.8	60.9	0.9 (0.7–1.3)	0.717	0.8 (0.6–1.2)	0.319
Adenosquamous carcinomas	80.0	53.3	53.3	53.3	1.3 (0.3–5.3)	0.706	1.4 (0.3-6.0)	0.627
Others (include neuroendocrine tumors, melanoma, germ cell tumor)	83.3	58.9	50.5	50.5	1.4 (0.7–2.9)	0.317	1.0 (0.5-2.0)	0.917

The overall observed 5-year survival rate in this study was 59.5%, which is relatively low than that reported in a previous nationwide study [19], but higher than the rate observed in a single-centre, single-ethnic group (Malay) study conducted at Hospital Universiti Sains Malaysia (HUSM) [26]. Compared to a meta-analysis of survival rate of cervical cancer in Asian countries, our study portrayed superior 5-year survival rate compared to the overall 5-year Malaysian cervical cancer survival rate of 38.2% [30]. Our data is comparable to that reported in other LMICs such as India (59.85%) [30]. Ethnicity and FIGO staging remain significant determining factors of cervical cancer survival rate.

Our study presents comprehensive data from the largest state of Malaysia, serviced by a single tertiary government hospital– Sarawak General Hospital– which is able to provide surgery, radiotherapy, chemotherapy and palliative care services. All diagnosed patients are referred to Sarawak General Hospital for their cancer treatment plan [31]. Around 40% of the Sarawak population live over 100 km from urban towns and cities. Additionally, 60% of the patients in this study belonged to the bottom 40% (B40) income group, defined as households with a monthly income of less than RM 5249 (USD 1186). Despite these challenges, the median duration from initial diagnosis to receiving first treatment was 1.8 months, while median time from referral (internal or external) to first being seen at Radiotherapy Unit (RTU) was 0.3 months (Table 2), indicating an efficient cancer care delivery system. The median follow-up duration time calculated from diagnosis to the last recorded visit date at RTU, was 9.3 months, of which during the follow up, treatment may have been completed for radically treated patients, and may or may not have been continued for palliative patients. On the other hand, 5% of our patients did not have any treatment recorded, indicating lost to follow-up or potential non-compliance to treatment. However, due to the absence of an integrated electronic medical record (EMR) system, we were unable to collect comprehensive data on duration of onset of cervical cancer symptoms, treatment completion rate, reason for non-compliance, response to treatment and cancer specific survival for all patients. This highlights the urgent need for an EMR system to establish a more comprehensive and accurate database.

**Table 2:** Characteristics of cervical cancer patients in Sarawak (*n* = 555)

Characteristics	Value
**Age category, n(%)**	
<30 years old	11 (2.0)
30–39 years old	69 (12.4)
40–49 years old	136 (24.5)
50–59 years old	159 (28.6)
≥ 60 years old	180 (32.4)
**FIGO cancer staging upon diagnosis**	
I	63 (11.4)
II	185 (33.5)
III	179 (32.4)
IV	126 (22.8)
**Duration of follow up from diagnosis (months), median**	27.1 (Range: 0.2–77.7)
Diagnosis to death (months) (*n* = 549)	Stage IV**: 23.6 (Range: 11.1–36.1)
Diagnosis to receiving treatment (months) (*n* = 523)	1.7 (Range: 0-38.5)
Referral to First seen at RTU (months) (*n* = 282)	0.3 (Range: 0-3.2)
First seen at RTU to receiving treatment (months) (*n* = 473)	0.5 (Range: 0-24.9)
Symptoms onset to diagnosis (weeks) (*n* = 177)	8.0 (Range: 1-182)
**Race, n(%)**	
Malay	103 (18.6)
Chinese	123 (22.2)
Iban	201 (36.1)
Bidayuh	39 (7.0)
Melanau	23 (4.1)
Others	68 (12.2)
**HPV Vaccination**	
No	6 (1.1%)
Yes	0 (0%)
Unknown	549 (98.9%)
**Marital Status, n(%)**	
Married	464 (83.6%)
Widowed	49 (8.8%)
Divorced	18 (3.2%)
Single	20 (3.6%)
Missing	4 (0.8%)
**Symptoms, n(%)**	
Post Menopausal Bleed	224 (40.4)
Intermenstrual Bleed	163 (29.4)
Abnormal Vaginal Discharge	36 (6.5)
Post Coital Bleed	19 (3.4)
Abdominal Pain	73 (13.2)
Abnormal Smear	12 (2.2)
**Total All Cause Deaths**	186 (33.6)
**WHO Histology**	
Squamous cell carcinomas	409 (73.7)
Adenocarcinomas	123 (22.2)
Adenosquamous carcinomas	5 (0.9)
Others (include neuroendocrine tumours, melanoma and germ cell tumours)	18 (3.2)
**Treatment**	
No treatment recorded	28 (5.0)
Chemotherapy alone	48 (8.6)
Radiation alone	109 (19.5)
Chemoradiation	316 (57.1)
Surgery +- Chemotherapy +- Radiation	53 (9.4)

**Out of thefour stages upon diagnosis, only Stage IV achieved median survival

Out of the 555 patients, we were able to trace 177 who had a recorded duration of symptoms. For these patients, the median duration from symptom onset to initial diagnosis was 8 months.

These relatively long duration of symptom onset along with advanced stage at diagnosis indicate a need for improved cervical health literacy among communities in Sarawak, consistent with previous studies conducted across Malaysia over the past decade [14, 32–34]. Many Malaysians are unaware that cervical cancer is preventable through regular screening, that the early stages of cervical cancer are typically asymptomatic, and that currently non-sexually active individuals are also at risk for the disease.

The World Health Organisation (WHO) cervical cancer elimination strategy has set a 90-70-90 target: by the end of 2030, 90% of girls should be vaccinated against HPV by the age of 15 years; 70% of all women aged 30–49 years should have at least two high-precision screening tests, 10 years apart; and 90% of screen-positive women, as well as 90% of women diagnosed with cancer, should receive treatment [35]. Malaysia launched a successful school-based HPV vaccination programme for girls aged 12–15 between 2010 and 2020. Currently, the public sector cervical screening programme in Malaysia targets women aged 30–65 and offers either an HPV DNA test every five years or a Pap smear every two to three years. The Malaysian Ministry of Health, Ministry of Women, Family and Community Development and Non-governmental Organisations (NGO) such as Program ROSE [36] have doubled national cervical screening uptake via innovative approaches such as self-sampling for HPV DNA testing, increasing it from 12.8% of eligible women in 2011 to 26% in 2020 [37]. However this still falls significantly short of the WHO target of 70%. Furthermore, although Sarawak reports one of the highest incidences of cervical cancer nationwide, local data on high-risk HPV (hrHPV) oncotypes and cervical cancer risk prediction remain limited and poorly understood [7] with most existing data derived from West Malaysian cohorts.

## Conclusion

This study underscores the need for a comprehensive, multipronged strategy for the prevention and control of cervical cancer among Sarawakian women. Key components of this approach include the effective implementation of HPV vaccination, the establishment of population-based cervical cancer screening and the development of culturally tailored cervical health literacy for ethnically diverse populations. Additionally, addressing socioeconomic barriers such as poverty and cultural stigma are critical.

Tackling these factors could substantially improve equitable access to HPV vaccination and screening as part of a cervical cancer elimination strategy in Sarawak. Further research is needed to study the biological and socio-economic determinants of cervical cancer risk in Sarawak’s ethnically diverse population.

Findings from this study also support the implementation of a multidisciplinary approach to cervical cancer prevention and management, as well as the establishment of a prospective state wide cervical cancer registry to accurately capture patient characteristics, patterns of care and survival outcomes in order to inform national cancer policies and service development.

### Limitation

This was a single-centre study without EMR. The data were collected from paper-based case notes with a substantial amount of missing data. Due to geographical and socioeconomic challenges, the death records for this study may not have been accurately reported by patient next-of-kin, hence skewing the 5-year survival rate in this study. Patient co-morbidities, treatment compliance, cause of death and cancer specific survival were not always available. Furthermore, the findings may not be generalisable to other populations due to the study being conducted in a single referral public health facility in Sarawak.

## Data Availability

The dataset generated and/or analysed during the current study are available in the [BMC Cervical Cancer Dataset MLSH] repository, [https://www.kaggle.com/datasets/melissalimsiawhan/bmc-cervical-cancer-dataset-mlsh].
